# Predictors of Long-Term Prognosis Focused on Kidney Function in Patients with Chronic Coronary Syndrome

**DOI:** 10.3390/diseases14020078

**Published:** 2026-02-19

**Authors:** Katarzyna Charkiewicz-Szeremeta, Emilia Sawicka-Śmiarowska, Marlena Dubatówka, Małgorzata Knapp, Klaudia Mickiewicz, Jacek Jamiołkowski, Andrzej Raczkowski, Marcin Kondraciuk, Anna Szpakowicz, Katarzyna Ptaszyńska, Karol A. Kamiński

**Affiliations:** 1 Department of Population Medicine and Lifestyle Diseases Prevention, Medical University of Bialystok, 15-269 Bialystok, Poland; katarzyna.charkiewicz006@gmail.com (K.C.-S.);; 2 Department of Cardiology and Internal Medicine, Center of Postgraduate Medical Education, Grochowski Hospital, 04-073 Warszawa, Poland; 3 Department of Cardiology and Internal Diseases, Medical University of Bialystok, 15-276 Bialystok, Poland

**Keywords:** chronic coronary syndrome, urine albumin/creatin ratio, eGFR, survival in CCS, KDIGO classification

## Abstract

Background: The number of patients with chronic coronary syndromes (CCS) is growing, influenced by factors such as increasing life expectancy and prevalence of risk factors. Thus, cardiovascular (CV) disease remains the leading cause of mortality and morbidity worldwide. The main objective of the study was to identify factors associated with long-term survival in patients with chronic coronary syndrome, with a focus on kidney function described by eGFR and albuminuria (assessed by uACR). Methods: The study comprised a total of 257 patients from Bialystok (Poland), aged ≤ 80 years, who 6–18 months earlier were hospitalized for acute coronary syndrome or elective myocardial revascularization. During the 80-month follow-up, 40 (15.6%) patients died, while there was no information about three (1.2%) patients. Patients with preserved eGFR and without albuminuria were characterized by the longest survival, with deterioration of prognosis in groups of progressive kidney dysfunction as defined by KDIGO based on eGFR and uACR. The primary endpoint was death from any cause. Results: Those who survived the 80-month follow-up period were younger (*p* < 0.001), had a lower waist circumference (*p* = 0.028), higher diastolic blood pressure (*p* = 0.026), lower NTproBNP (*p* < 0.001) and hsCRP (*p* = 0.001) concentrations, reduced eGFR (*p* = 0.004) and increased ACR (*p* = 0.023) were strongly associated with mortality. In logistic regression analysis with stepwise elimination of variables, the strongest factors affecting survival were hemoglobin concentration, left ventricle ejection fraction (LVEF) and hsCRP. Conclusions: Measurement of albuminuria, in addition to eGFR, allows patients to be correctly classified into CV risk categories and facilitates appropriate treatment of patients with CCS. Higher diastolic blood pressure (but still within normal range) was found in patients who later survived 6 years. Measurements of hsCRP, hemoglobin concentration and LVEF help to identify CCS patients at the highest risk of mortality in long-term follow-up.

## 1. Introduction

The prevalence of cardiovascular disease is increasing worldwide, reaching pandemic proportions. Coronary artery disease is a leading cause of death and morbidity in many countries [1]. Despite recent progress in its diagnostics and treatment, we still need better tools for risk stratification and better strategies based on comorbidities.

A typical presentation of coronary artery disease (CAD) is chronic coronary syndrome, which affects between 5% and 12% of the population (according to various definitions) over the age of 45 [2]. The concept of chronic coronary syndrome (CCS) is a response to the revision of the approach to the disease in relation to pathophysiology. The nomenclature was introduced as a replacement for stable ischemic heart disease and expanded to include a classification based on six scenarios [3], which seems to be practical in daily medical practice both diagnostically and therapeutically.

The number of patients with CCS is likely to go up due to increasing life expectancy, rising prevalence of diabetes and improved survival of patients with acute coronary syndrome (ACS) [4,5,6]. Although significant advances in the diagnosis and pharmacological and invasive treatment of CAD have been implemented in clinical practice in recent decades, the number of studies on the management of patients with CCS still has room for improvement [7].

One of the key comorbidities that shape the development, progression as well as prognosis in chronic coronary syndromes is impaired kidney function, which should be looked at not merely as decreased eGFR, but as a complex of metabolic, neurohormonal and hemodynamic disturbances that could be predicted based on both eGFR and albuminuria [8].

The main objective of our study was to determine the clinical factors related to long-term survival in patients with chronic coronary syndrome, with special attention to the potential impact of eGFR and albuminuria. The primary endpoint of our study was death from any cause.

## 2. Methods

### 2.1. Study Population

Patients with CCS between the ages of 41 and 79 were included in the study conducted between 2016 and 2018 as part of the project POLASPIRE (Polish Action on Secondary and Primary Prevention by Intervention to Reduce Events) [8,9]. A total of 257 individuals were admitted to the Provincial and University Hospitals in Bialystok due to elective percutaneous coronary intervention (PCI) 96 (37.35%) or acute myocardial infarction with ST-segment elevation (STEMI) 65 (25.29%) or acute myocardial infarction with non-ST-segment elevation (NSTEMI) 71 (27.63%) or unstable angina 25 (9.73%) that had occurred 6 to 18 months prior to the study visit. In the group, there were 190 men (73.93%). In the 80-month follow-up, the deaths of 40 (15.56%) subjects were reported, and there was no information about three (1.17%) patients (detailed information in Figure S1). Missing patients were not included in the statistical analyses. The numbers in Figure S3 represent the prevalence in the examined population. Over six-year survival was assessed based on the telephone follow-up.

### 2.2. Data Collection and Assays

A comprehensive assessment was performed. Questionnaires obtained information about the participants’ medical histories during the research visit. Every research patient underwent a physical examination as well as a laboratory assessment. Peripheral intravenous blood samples were obtained following an overnight fast. The oral glucose tolerance test (OGTT) was administered to the individuals who reported being free of diabetes. Blood samples were collected once during a visit 6 to 18 months after the incident. Urine Albuminuria/Creatine Rate (uACR) was assayed using urine samples obtained at random. All samples were analyzed in the local laboratory of the University Hospital in Bialystok. The anthropometric measurements, including weight, height, circumferences of the waist, abdomen, and hips, were taken by qualified medical personnel. The formula for calculating body mass index (BMI) was weight in kilograms divided by height in square meters. The oscillometric technique was used to measure each participant’s blood pressure (BP) after sitting for at least five minutes. An electrocardiography (ECG) was taken while the patient was at rest. The 2009 Chronic Kidney Disease Epidemiology Collaboration (CKD-EPI) algorithm was used to determine the estimated Glomerular Filtration Rate (eGFR). In our study, the KDIGO classification was found to be based on eGFR and uACR from a single visit with a random urine sample.

To assess over 6-year survival, some of the patients were invited to the hospital and others were contacted by telephone.

### 2.3. Ethical Issues

The Ethics Committee of the Medical University of Bialystok provided ethical approval for this study (approval numbers R-I-002/323/2016).

The study was conducted in accordance with the Declaration of Helsinki.

All participants provided written informed consent.

### 2.4. Statistical Analysis

Statistical analysis was performed using IBM SPSS Statistics 26.0 (Armonk, NY, USA). The results were regarded as statistically significant at the 0.05 level.

The normality of distribution was verified using the Kolomogorow–Smirnow test. The parameters of the normal distribution were the mean and standard deviation (SD). For non-normal distribution, continuous variables were shown as the median with the interquartile range (IQR) [first quartile–third quartile]. Variables with normal distribution were analyzed using ANOVA. Continuous variables other than normal were compared using the Mann–Whitney U test. χ^2^ test was used to analyze qualitative data. Models for univariate and multivariate logistic regression were developed to identify factors determining survival. A Wald stepwise backward logistic regression of factors significantly associated with survival was used. The Kaplan–Meier curves were plotted to show survival in different cardiovascular risk groups over six years following the survey. Cox regression was used to assess the interaction between KDIGO class and survival, also to find factors associated with mortality. Receiver operating characteristic (ROC) curves were plotted based on the relevant data in the comparative analysis of the deceased group and the surviving group. Single-factor ROC curves for median observation time with time-dependent AUC were performed to assess the predictive power of the parameters. Frequency tables were used to summarize categorical variables.

## 3. Results

The study population’s baseline characteristics are shown in Table 1. The study sample presented a mean age of 64.38 years (SD 8.14). Overall, the median of eGFR was 77.19 (65.79–90.7) and the median of uACR was 5.14 (0.00–12.85).

72.76% of the sample consisted of patients without severe renal illness, while 18.68% noted mild CKD. Moderate CKD was present in 7.4% of patients. Severe CKD was observed in 1.17% of the research participants. A more detailed classification of patients into groups according to their eGFR and uACR can be found in Tables S1–S3 in the Supplementary Material.

**Figure 1:**
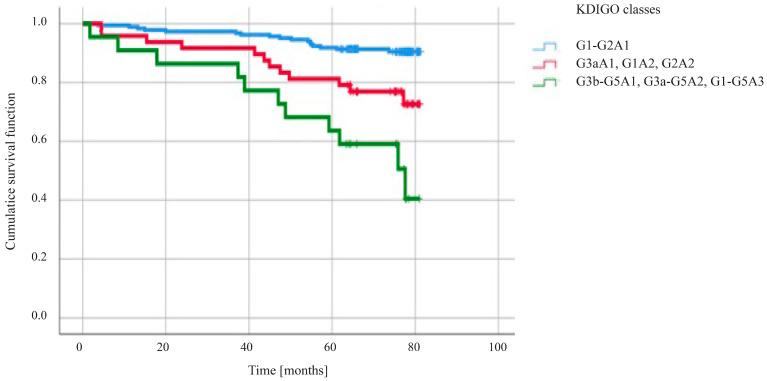
Kaplan–Meier curve showing survival by risk category according to the KDIGO classification. 80-month observation as a maximum follow-up (*p* values < 0.001).

Figure 1 shows the survival difference (*p* < 0.001) between patients grouped according to the impairment of kidney function according to KDIGO: severe, moderate and mild risk (Figure S3). The best survival can be expected in patients from the mild or no CKD group. In contrast, patients with the severe risk group are characterized by the greatest mortality; mortality in this group is the highest relative to other groups. The group with moderate risk according to CKD is a group at intermediate risk of death. Based on Cox regression and post hoc test, the interaction between groups was determined; i.e., between the mild or no CKD group and the moderate CKD risk group, *p* = 0.004. Between the moderate CKD risk group and the severe disease group, *p* = 0.045. The comparison between the mild or no CKD group and the severe CKD group yielded *p* < 0.001.

The population of subjects who survived was compared with the population of subjects who died in Table 2. Those who survived the 80-month follow-up period were younger (*p* < 0.001), had a lower waist circumference (*p* = 0.028). Higher diastolic blood pressure (*p* = 0.026) had a significant effect on survival. In peripheral blood count, a predictor of mortality was lower: RBC (*p* < 0.001), HGB (*p* < 0.001), HCT (*p* < 0.001) and higher RDW CV (*p* = 0.001). Mortality was positively associated with lower eGFR (*p* = 0.004) and elevated uACR (*p* = 0.023). Significantly lower levels of NTproBNP (*p* < 0.001) and hsCRP (*p* = 0.001) concentration were observed in the survivors group. Individual lipid profile components did not significantly affect survival in the study population. Survival was negatively affected by the history of diabetes (*p* = 0.032); in addition, a higher percentage of hemoglobin A1c (HbA1c) was also observed in patients who died during follow-up (*p* = 0.020).

The ROC curves showed factors significantly affecting survival rates (Figure S2). The supplement includes graphs. The ROC curves included in the supplement contain factors that have been shown to be statistically significant for mortality. Thus, the strongest predictors of death are EF, HGB and age. RDW-CV, BPd, HbA1c, eGFR, and uACR may also be markers of worsening survival and serve as auxiliary indicators in identification.

In univariate logistic regression analysis, the variables that remained associated with survival in the Unadjusted Model were waist circumference, LVEF, BPd, and peripheral blood count parameters such as RBC, HGB, HCT, and RDW CV (Table 3). In addition, NT pro-BNP and hsCRP concentrations were significantly associated with survival in biochemical tests.

In Cox regression analysis the strongest factors associated with survival were age, HGB, LVEF and hsCRP. (Table 4).

## 4. Discussion

This paper focuses on the impact of multiple factors on long-term mortality in chronic coronary syndromes, specifically considering kidney function based on the KDIGO classification and the role of uACR in this risk stratification. Cardiologists often focus primarily on eGFR, omitting assessment of urinary albuminuria. As our study shows, the ACR should not be overlooked. First of all, looking only at eGFR, one may assume that the population of relatively healthy patients is larger than that after correcting for the uACR. This erroneous assumption may result in an underestimation of the actual number of patients with a significantly higher cardiovascular risk and, therefore, higher mortality. As shown in Figure 1, the correct classification into a KDIGO group, which is based on eGFR and uACR criteria, is crucial in the prediction of long-term mortality. Although uACR as an individual factor did not come out statistically significant in univariate analysis, its inclusion as a component of each group in the KDIGO classification (G1-G2A1 vs. other classes) shows statistical significance. Our findings indicate that the higher the albuminuria and the lower the eGFR, the lower the 6-year survival rate.

### 4.1. Survival of the Population with CCS

Based on the available large studies, we can approximate the mortality statistics for the patient group. During the 6-month follow-up, 2.6% of patients died in the ESC pilot registry [10]. In the REACH registry, the overall mortality rate was 2.8% per year in the subgroup of patients diagnosed with cerebrovascular disease [10]. Parma et al. compared the long-term results of the Polish and European populations registered in the CLARIFY registry. The rate of death from all causes over 5 years among Polish and European patients was 8.5% and 7.9%, respectively [11]. The 5-year mortality rate in the entire PRESAGE registry population was 15.3% [7].

In our 6-year follow-up, we observed similar survival results to those reported in the PRESAGE trial, namely, the mortality rate in our research was 15.75% (Table 2). Moreover, classifying patients according to KDIGO presented a good way to evaluate prognosis as greater the severity of CKD have higher mortality. (Figure 1).

### 4.2. Relationship of KDIGO Classes on Mortality in the CCS Patient Population

Combining eGFR with uACR allowed more precise allocation to risk groups, allowing us to place them on a specific treatment regimen. In this publication, we aimed to identify patients with preserved eGFR, in whom increased uACR places them in a higher risk group. In this way, we distinguished a group of patients in whom CKD diagnosis may be easily “overlooked” in daily diagnosis. CBC parameters, i.e., RBC, RDW CV, as well as biochemical parameters, i.e., iron, sodium, hsCRP, NT proBNP, glucose and HbA1c, as well as HR identified patients with normal eGFR who were reclassified to a higher risk group by uACR.

A different Diabetes Heart Study found that renal function and albuminuria were independent risk factors for all-cause mortality and CVD mortality in American Europeans in a subgroup of people with type 2 diabetes [12].

However, looking at all categories in the KDIGO classification and comparing them against each other, we get a clear picture of the effect of KDIGO class on the survival of CCS patients. In univariable analysis we found that patients with preserved eGFR and no overt albuminuria (G1-G2A1–low risk group) had better prognosis than other patients with CCS (Table 3). The above data demonstrate how important it is to correctly classify a patient to assess their risk early in the treatment process. The implementation of a routine assessment of albuminuria should help assign the patient to a CVD risk group and increase the vigilance of the diagnostician in the treatment process. These claims should not be undermined by the lack of a statistically significant effect of uACR in the final model of the Cox regression analysis, in which we included several variables strictly associated with KDIGO class, namely age, hsCRP, HGB and LVEF. They may be good (even better than eGFR) markers of the poor prognosis, but it is kidney function (combined eGFR and uACR) that fully explains this phenomenon and provides an important treatment target in certain patients.

### 4.3. Impact of Other Factors on Survival in CCS Patients

We observed that the RBC system is related to survival in CCS patients. Based on Gyeongsil Lee’s study of the general population reporting the effect of hemoglobin concentration on mortality, it demonstrated an association between hemoglobin concentration and cardiovascular mortality in both men and women after accounting for CVD risk factors [13]. In our study, relying on logistic regression with an unadjusted model also established the negative impact of reduced HGB levels on survival. Moreover, these data strengthened the results of the Cox regression used in our study, which indicated a relationship at the level of *p* = 0.003 in the final model (Table 4).

Our study also confirms findings of a meta-analysis of Su et al., which indicated higher levels of RDW are associated with an increased risk of mortality in patients with established CAD [14].

Blood pressure

It is also worth mentioning the observed impact of BPd on survival. As is widely known, in patients with CCS, pharmacotherapy for hypertension should be optimized until normal blood pressure values are achieved. However, our study of a group of CCS patients based on CKD parameters shows that blood pressure should be considered as a factor that can have an indirect effect, but also as a sensitive factor that is influenced by many indirect factors. Nevertheless, this highlights the need to pay more attention to BPd values and isolated diastolic hypertension.

Abdominal obesity

Abdominal obesity is an established risk factor of cardiovascular disease. Recently published analysis suggests that increased waist circumference is also related to increased mortality in patients with CAD [15,16]. Also, based on our results, we can confirm the importance of waist circumference measurement in establishing mortality risk in patients with CCS.

Biochemical markers

There are several publications describing the role of NT-proBNP in the prognosis of patients with CCS. Kragelung et al. presented [17] NT-pro-BNP as a marker of long-term mortality in patients with stable coronary disease and provided prognostic information above and beyond that provided by conventional cardiovascular risk factors and the degree of LVEF. Our study also showed a positive association of high NT-proBNP concentration and mortality in the CCS population (Table 3).

The inflammatory marker-serum C-reactive protein (CRP) is associated with the development and progression of atherosclerosis, hence adversely affecting mortality [18]. Our study showed an association of low-grade chronic inflammation, as diagnosed by mildly elevated CRP concentrations (measured using high-sensitivity assay—hsCRP) with adverse prognosis in patients with CCS. Moreover, we see a clear association of the elevated CRP with increased uACR.

High levels of hsCRP are an independent risk linked to long-term mortality among patients with CAD, according to Huang’s study on patients with stable coronary artery disease, which also supports the aforementioned analyses [19].

Other prognostic markers

Imaging techniques may provide additional prognostic value. Several non-invasive imaging techniques are currently available to assess the presence and extent of viable myocardium, including myocardial viability on CMR, which has shown prognostic value in patients with CCS [20].

In addition, the use of advanced echocardiography (e.g., GLS) and exercise testing is also important in improving prognosis and better determining the burden of CAD [21].

### 4.4. Study Limitations

The data included in our analysis are limited to total mortality; we were not able to precisely determine the cause of death in all patients. Moreover, the number of patients examined is relatively small, which prevents detailed model comparisons—particularly reclassification and discrimination statistics. In addition, the study did not include patients over the age of 80. Therefore, our conclusions should be verified in further studies.

## 5. Conclusions

(1) The long-term risk of death in CCS patients may depends on the stage of chronic kidney disease, which requires the assessment of albuminuria.
(2) LVEF, hsCRP, and HGB were independently associated with long-term risk of death in the CCS patient population.

## Figures and Tables

**Table 1:** Baseline characteristics of the study population.

Variables	Value (*n* = 257)
Age, years	64.38 ± 8.14
Gender, male	190 (73.93)
Weight, kg	85.41 ± 16.4
Waist circumference, cm	99.33 ± 11.79
HR, bpm	65 (58–72.25)
BPs, mmHg	131.5 (120.75–147)
BPd, mmHg	83.62 ± 10.94
eGFR, mL/min/1.73 m^2^	77.19 (65.79–90.7)
WBC, thou/μL	6.3 (5.3–7.5)
RBC, mln/μL	4.69 (4.35–4.92)
HGB, g/dL	13.85 ± 1.22
RDW CV, %	14.5 (13.9–15.5)
MCV, fL	86.95 (84.03–89.9)
Serum iron concentration, μg/dL	98.1 (81.08–122.88)
Serum sodium, mmol/L	138.1 (136.7–140.1)
Serum potassium, mmol/L	4.28 (4.06–4.57)
Serum chloride, mmol/L	103.01 ± 2.9
hsCRP, mg/L	1.09 (0.54–2.48)
Serum calcium, mmol/L	2.41 (2.36–2.47)
Serum inorganic phosphate, mmol/L	3.13 (2.82–3.55)
Total cholesterol, mg/dL	153 (128–180)
LDL, mg/dL	83.8 (68.8–105.45)
HDL, mg/dL	48 (41–61)
Triglyceride, mg/dL	103 (73.25–154.75)
Fasting Glucose, mg/dL	105 (96.8–121.5)
uACR, mg/g	5.14 (0–12.85)
HbA1c, %	5.85 (5.6–6.3)
NT proBNP, pg/mL	170.75 (86.9–402.05)
Pulse pressure, mmHg	49.5 (40–58)
Diabetes, *n*	102 (39.7)
Hypertension, *n*	207 (80.5)
ACE-inhibitors, *n*	165 (64.2)
ARB, *n*	5 (1.9)
MRA, *n*	79 (30.7)
SGLT2 inhibitors, *n*	2 (0.8)

Data are presented as median (Q1–Q3) or *n* (%) or mean ± SD. Q1, quartile 1; Q3, quartile 3; SD, standard deviation; kg, kilogram; cm, centimeter; HR, heart rate; bpm, beats per minute; BPs, systolic blood pressure; BPd, diastolic blood pressure; mmHg, millimeters of mercury; eGFR, estimated glomerular filtration rate Chronic Kidney Disease Epidemiology Collaboration Equation; mL, milliliter; min, minute; m^2^, square meter; WBC, White Blood Cells; thou, thousand; μL, microliter; RBC, Red Blood Cells; mln, million; HGB, hemoglobin; g, gram; dL, deciliter; RDW CV, Red Cell Distribution Width in%; MCV, Mean Corpuscular Volume; fL, femtoliter; μg, microgram; mmol, millimole; L, Liter; hsCRP, high-sensitivity C-reactive protein; mg, milligram; LDL, Low-Density Lipoprotein; HDL, High-Density Lipoprotein; uACR, Urine Albumin/Creatinine Ratio; HbA1c, Glycated hemoglobin; NT-proBNP, N-terminal pro-brain natriuretic peptide; pg, picogram; ACE-inhibitors, Angiotensin-Converting Enzyme Inhibitors; ARB, Angiotensin II Receptor Blockers; MRA, Mineralocoticoid Receptor Antagonists; SGLT2 inhibitors, Sodium-Glucose Cotransporter-2.

**Table 2:** Study population characteristics according to survival.

Study Population (*n* = 254)			
Variables	Survivors *n* = 214	All-Cause Dead *n* = 40	*p* Values
Age, years	63.45 ± 7.92	70.40 ± 5.76	<0.001
Gender, male	160 (74.77)	29 (72.5)	0.764
Weight, kg	85.60 ± 16.29	84.86 ± 17.66	0.797
Waist circumference, cm	98.71 ± 11.72	103.18 ± 11.72	0.028
HR, bpm	65.00 (58.00–71.00)	68.00 (60.25–74.50)	0.222
BPs, mmHg	132.00 (122.00–147.00)	131.00 (117.50–147.00)	0.677
BPd, mmHg	84.37 ± 10.77	80.13 ± 11.47	0.026
eGFR, mL/min/1.73m^2^	77.70 (67.43–90.84)	66.18 (45.38–88.26)	0.004
WBC, tys/μL	6.30 (5.30–7.50)	6.45 (5.23–7.93)	0.786
RBC, mln/μL	4.74 (4.45–4.97)	4.28 (4.04–4.71)	<0.001
HGB, g/dL	14.04 ± 1.10	12.86 ± 1.37	<0.001
HCT, %	41.10 (38.90–43.20)	38.00 (35.43–40.15)	<0.001
RDW CV, %	14.50 (13.80–15.30)	46.50 (44.20–50.83)	0.001
PLT, tys/μL	215.00 (185.50–251.50)	218.00 (172.25–255.00)	0.478
MCV, fL	86.50 (83.95–89.85)	88.05 (84.13–89.98)	0.324
Serum iron concentration, μg/dL	99.95 (82.45–124.38)	88.10 (79.80–117.40)	0.110
Serum sodium, mmol/L	138.00 (136.65–140.10)	138.10 (136.40–140.20)	0.742
Serum potassium, mmol/L	4.29 (4.06–4.57)	4.24 (3.97–4.56)	0.757
Serum chloride, mmol/L	102.99 ± 2.85	102.84 ± 3.19	0.799
hsCRP, mg/L	1.01 (0.50–2.28)	1.98 (0.73–6.80)	0.001
Serum calcium, mmol/L	2.41 (2.35–2.47)	2.40 (2.36–2.47)	0.566
Serum inorganic phosphate, mmol/L	3.14 (2.87–3.56)	3.06 (2.79–3.49)	0.438
Total cholesterol, mg/dL	153.00 (128.75–180.25)	158.00 (128.00–182.00)	0.939
LDL, mg/dL	84.35 (67.58–104.40)	81.70 (70.80–110.00)	0.821
HDL, mg/dL	48.50 (41.00–61.00)	46.00 (36.00–60.00)	0.263
Triglyceride, mg/dL	103.00 (73.75–156.25)	103.00 (66.00–154.00)	0.760
Fasting glucose, mg/dL	104.00 (96.10–118.00)	110.00 (99.00–128.00)	0.301
uACR, mg/g	4.94 (0.00–9.79)	9.86 (0.56–22.27)	0.023
HbA1c, %	5.80 (5.60–6.20)	6.10 (5.80–6.60)	0.020
NT proBNP, pg/mL	140.45 (83.51–333.53)	708.30 (200.80–1539.00)	<0.001
Pulse pressure, mmHg	49 (39–59)	49.5 (42.25–56.75)	0.939
Diabetes, *n*	79 (36.9)	22 (55.0)	0.032
LVEF, %	52.7 (47.8–58.1)	48.0 (36.9–51.5)	<0.001
Hypertension, *n*	173 (80.8)	32 (80.0)	0.537
ACE-inhibitors, *n*	138 (64.5)	25 (62.5)	0.493
ARB, *n*	3 (1.4)	2 (5.0)	0.139
MRA, *n*	59 (27.6)	18 (45.0)	0.022
SGLT2 inhibitors, *n*	2 (0.9)	0	0.534

Data are showed as median (Q1–Q3) or *n* (%) or mean ± SD. Q1, quartile 1; Q3, quartile 3; SD, standard deviation; kg, kilogram; cm, centimeter; HR, heart rate; bpm, beats per minute; BPs, systolic blood pressure; BPd, diastolic blood pressure; mmHg, millimeters of mercury; eGFR, estimated glomerular filtration rate Chronic Kidney Disease Epidemiology Collaboration Equation; mL, milliliter; min, minute; m^2^, square meter; WBC, White Blood Cells; μL, microliter; RBC, Red Blood Cells; mln, million; HGB, hemoglobin; g, gram; dL, deciliter; RDW CV, Red Cell Distribution Width in%; PLT, Platelet Blood Test; MCV, Mean Corpuscular Volume; fL, femtoliter; μg, microgram; mmol, millimole; L, Liter; hsCRP, high-sensitivity C-reactive protein; mg, milligram; LDL, Low-Density Lipoprotein; HDL, High-Density Lipoprotein; uACR, Urine Albumin/Creatinine Ratio; HbA1c, Glycated hemoglobin; NT-proBNP, N-terminal pro-brain natriuretic peptide; pg, picogram; LVEF, Left Ventricle Ejection Fraction; ACE-inhibitors, Angiotensin-Converting Enzyme Inhibitors; ARB, Angiotensin II Receptor Blockers; MRA, Mineralocoticoid Receptor Antagonists; SGLT2 inhibitors, Sodium-Glucose Cotransporter-2.

**Table 3:** Univariable predictors of mortality.

Variables	Unadjusted Model	
	OR (95% CI)	*p* Values
Age, years	1.145 (1.083;1.211)	<0.001
Gender, male	0.890 (0.416;1.902)	0.763
LVEF, %	0.913 (0.878;0.950)	<0.001
BMI, kg/m^2^	1.027 (0.964;1.094)	0.409
Waist circumference, cm	1.034 (1.003;1.065)	0.030
HR, bpm	1.018 (0.986;1.050)	0.268
BPs, mmHg	0.997 (0.981;1.015)	0.771
BPd, mmHg	0.962 (0.930;0.996)	0.027
Total cholesterol, mg/dL	0.999 (0.991;1.007)	0.825
LDL, mg/dL	1.000 (0.991;1.010)	0.919
HDL, mg/dL	0.989 (0.967;1.012)	0.358
Triglyceride, mg/dL	1.000 (0.997;1.003)	0.783
Fasting glucose, mg/dL	1.001 (0.991;1.012)	0.802
eGFR, mL/min/1.73 m^2^	0.964 (0.946;0.983)	<0.001
uACR, mg/g	1.001 (0.998;1.004)	0.593
HbA1c, %	1.219 (0.915;1.621)	0.174
NT-proBNP, pg/mL	1.002 (1.001;1.002)	<0.001
WBC, tys/μL	1.013 (0.839;1.222)	0.895
RBC, mln/μL	0.215 (0.092;0.505)	<0.001
HGB, g/dL	0.423 (0.302;0.591)	<0.001
HCT, %	0.768 (0.686;0.860)	<0.001
RDW CV, %	1.438 (1.146;1.803)	0.002
PLT, tys/μL	0.997 (0.997;1.003)	0.345
MCV, fL	1.033 (0.970;1.098)	0.311
hsCRP, mg/L	1.208 (1.083;1.346)	0.001
KDIGO		
G1-G2A1 vs. other groups	4.807 (2.374;9.734)	<0.001

Data are showed OR, odds ratio; CI, confidence interval; kg, kilogram; cm, centimeter; HR, heart rate; bpm, beats per minute; BPs, systolic blood pressure; BPd, diastolic blood pressure; mmHg, millimeters of mercury; eGFR, estimated glomerular filtration rate Chronic Kidney Disease Epidemiology Collaboration Equation; kg, kilogram; mL, milliliter; min, minute; m^2^, square meter; WBC, White Blood Cells; thou, thousand; μL, microliter; RBC, Red Blood Cells; mln, million; HGB, hemoglobin; g, gram; dL, deciliter; RDW CV, Red Cell Distribution Width in%; PLT, Platelet Blood Test; MCV, Mean Corpuscular Volume; fL, femtoliter; mmol, millimole; L, Liter; hsCRP, high-sensitivity C-reactive protein; mg, milligram; LDL, Low-Density Lipoprotein; HDL, High-Density Lipoprotein; uACR, Urine Albumin/Creatinine Ratio; HbA1c, Glycated hemoglobin; NT-proBNP, N-terminal pro-brain natriuretic peptide; pg, picogram, LVEF, Left Ventricle Ejection Fraction.

**Table 4:** Results of Cox regression of factors associated with mortality.

Variables	Full Model		Final Model	
	HR (95% CI)	*p* Values	HR (95% CI)	*p* Values
Age, years	1.089 (1.027;1.155)	0.004	1.112 (1.037;1.193)	0.003
HGB, g/dL	0.607 (0.427;0.862)	0.005	0.652 (0.492;0.865)	0.003
hsCRP, mg/L	1.036 (1.000;1.073)	0.053	1.039 (1.016;1.062)	0.001
LVEF, %	0.932 (0.897;0.968)	<0.001	0.942 (0.914;0.971)	<0.001
NT-proBNP, pg/mL	1.000 (1.000;1.000)	0.618	-	
RDW CV, %	0.992 (0.852;1.156)	0.921	-	
Waist circumference, cm	1.023 (0.991;1.057)	0.163	-	
BPd, mmHg	0.972 (0.941;1.004)	0.084	-	
uACR in groups (A1–A3)	1.001 (0.997;1.005)	0.718	-	

HR, hazard ratio; CI, confidence interval; HGB, hemoglobin; g, gram; dL, deciliter; hsCRP, high-sensitivity C-reactive protein; mg, milligram; L, Liter; LVEF, Left Ventricle Ejection Fraction; NT-proBNP, N-terminal pro-brain natriuretic peptide; pg, picogram; mL, milliliter; RDW CV, Red Cell Distribution Width in %; cm, centimeter; BPd, diastolic blood pressure; mmHg, millimeters of mercury; uACR, Urine Albumin/Creatinine Ratio; A1–A3 groups, groups included in KDIGO classes.

## Data Availability

The data presented in this study are available on request from the corresponding author due to privacy restrictions.

## Supplementary Materials

The following supporting information can be downloaded at: https://www.mdpi.com/article/10.3390/diseases14020078/s1, Figure S1: Population distribution; Figure S2: Parameters of age, HGB, EF, BPd, eGFR, uACR, HbA1c and RDW CV presented on the ROC curve; Figure S3: ESC 2021 guideline on cardiovascular disease prevention in clinical practice risk class conversion from chronic renal disease risk classes; Table S1: Study population characteristics based on eGFR; Table S2: Study population characteristics based on uACR; Table S3: Comparison of groups of patients with absent and present albuminuria only with normal renal function.
