# Supplementary material for: Predictors of Long-Term Prognosis Focused on Kidney Function in Patients with Chronic Coronary Syndrome
Source: Diseases. 2026 Feb 19;14(2):78. doi: 10.3390/diseases14020078 (PMC12939443; doi:10.3390/diseases14020078)
Supplement: Supplementary file 1 [file diseases-14-00078-s001.zip › Table S1.pdf]

Table S1 Study population characteristics based on eGFR.

Study population (n=257)			
Variables	Subjects <60 (n=44)	Subjects ≥60 (n=212)	p Values
Age, years	71.86 ± 5.37	62.77 ± 7.73	<0.001
Gender, male	25 (56.82)	165 (77.83)	0.004
Weight, kg	83.90 ± 15.95	85.84 ± 16.47	0.476
Waist circumference, cm	100.94 ± 12.00	99.05 ± 11.76	0.332
HR, bpm	63.00 (58.00-74.00)	65.00 (58.75-72.25)	0.865
BPs, mmHg	134.00 (117.00-147.00)	131.00 (121.00-146.25)	0.789
BPd, mmHg	79.60 ± 9.97	84.52 ± 10.94	0.007
WBC, tys/µL	6.65 (5.30-7.45)	6.30 (5.20-7.50)	0.468
RBC, mln/µL	4.32 (4.11-4.74)	4.74 (4.45-5.03)	<0.001
HGB, g/dL	13.04 ± 1.09	14.02 ± 1.18	<0.001
HCT, %	38.05 (35.78-40.70)	41.20 (38.80-43.40)	<0.001
RDW CV, %	15.10 (14.03-15.70)	14.50 (13.80-15.40)	0.030
PLT, tys/µL	199.50 (171.50-235.00)	217.00 (186.00-255.00)	0.058
MCV, fL	88.35 (85.23-89.88)	86.40 (83.90-90.20)	0.186
Serum iron concentration, µg/dL	91.70 (70.20-112.23)	100.05 (82.33-124.73)	0.018
Serum sodium, mmol/L	137.90 (136.80-1440.20)	138.10 (136.60-140.00)	0.899
Serum potassium, mmol/L	4.37 (4.11-4.80)	4.28 (4.05-4.51)	0.150
Serum chloride, mmol/L	103.32 ± 2.83	102.94 ± 2.92	0.435
hsCRP, mg/L	1.49 (0.55-3.41)	1.03 (0.54-2.44)	0.175
Total cholesterol, mg/dL	151.00 (127.50-176.00)	153.50 (128.00-180.75)	0.867
LDL, mg/dL	84.40 (69.10-103.65)	83.40 (68.03-106.35)	0.996
HDL, mg/dL	45.50 (38.25-54.00)	49.00 (41.00-61.75)	0.115
Triglyceride, mg/dL	114.50 (74.25-167.50)	101.00 (72.25-154.00)	0.227

Fasting Glucose, mg/dL	104.50 (97.75-129.50)	105.00 (96.60-121.00)	0.578
uACR, mg/g	6.51 (0.00-15.17)	4.99 (0.00-11.28)	0.322
HbA1c, %	5.90 (5.70-6.60)	5.80 (5.60-6.20)	0.062
NT proBNP, pg/mL	538.85 (225.40-1243.50)	139.80 (80.63-325.35)	<0.001
Pulse pressure, mmHg	55 (44-61.75)	48 (38-57)	0.039
LVEF, %	50.61 (39.97-55.60)	52.16 (47.51-57.22)	0.091

Data are showed as median (Q1-Q3) or n (%) or mean ± SD. Q1, quartile 1; Q3, quartile 3; SD, standard deviation; kg, kilogram; cm, centimeter; HR, heart rate; bpm, beats per minute; BPs, systolic blood pressure; BPd, diastolic blood pressure; mmHg, millimeters of mercury; eGFR, estimated glomerular filtration rate Chronic Kidney Disease Epidemiology Collaboration *Equation*; mL, milliliter; min, minute; m^2, square meter; WBC, White Blood Cells; thou, thousand; µL, microliter; RBC, Red Blood Cells; mln, million; HGB, hemoglobin; g, gram; dL, deciliter; RDW CV, Red Cell Distribution Width in%; PLT, Platelet Blood Test; MCV, Mean Corpuscular Volume; fL, femtoliter; µg, microgram; mmol, millimole; L, Liter; hsCRP, high-sensitivity C-reactive protein; mg, milligram; LDL, Low-Density Lipoprotein; HDL, High-Density Lipoprotein; uACR, Urine Albumin/Creatinine Ratio; HbA1c, Glycated hemoglobin; NT-proBNP, N-terminal pro-brain natriuretic peptide; pg, picogram; LVEF, Left Ventricle Ejection Fraction.
