# Supplementary material for: Predictors of Long-Term Prognosis Focused on Kidney Function in Patients with Chronic Coronary Syndrome
Source: Diseases. 2026 Feb 19;14(2):78. doi: 10.3390/diseases14020078 (PMC12939443; doi:10.3390/diseases14020078)
Supplement: Supplementary file 1 [file diseases-14-00078-s001.zip › Table S2.pdf]

Table S2 Study population characteristics based on uACR.

Study population (n=194)			
Variables	Subjects <30 (n=172)	Subjects >30 (n=22)	p-value
Age, years	63.92 +- 7.76	66.68 +- 7.31	0.116
Gender, male	123 (71.51)	16 (72.73)	0.906
Weight, kg	85.50 ± 16.42	89.87 ± 19.61	0.252
Waist circumference, cm	98.78 ±11.90	104.16 ± 14.62	0.054
HR, bpm	64.00 (58.00-71.50)	68.00 (62.00-81.75)	0.021
BPs, mmHg	130.00 (120.00-144.50)	136.00 (126.50-149.00)	0.136
BPd, mmHg	82.99 ± 11.38	86.14 ±7.29	0.209
eGFR, ml/min/1.73m2	76.59 (66.18-90.64)	72.55 (61.20-86.44)	0.450
WBC, tys/µL	6.30 (5.30-7.50)	6.65 (4.88-8.45)	0.770
RBC, mln/µL	4.67 (4.37-4.94)	4.44 (4.06-4.82)	0.022
HGB, g/dL	13.90 ± 1.15	13.25 ± 1.55	0.035
HCT, %	40.85 (38.13-43.00)	39.05 (35.85-41.83)	0.111
RDW CV, %	14.40 (13.70-15.40)	15.20 (14.30-16.70)	0.029
PLT, tys/µL	212.50 (184.25-255.00)	219.00 (173.75-267.75)	0.851
MCV, fL	87.20 (83.93-89.96)	219.00 (173.75-267.75)	0.514
Serum iron concentration, µg/dL	100.50 (83.60-125.50)	84.30 (69.25-108.63)	0.030
Serum sodium, mmol/L	138.10 (136.60-140.10)	137.10 (135.95-138.35)	0.034
Serum potassium, mmol/L	4.29 (4.04-4.59)	4.29 (4.11-4.57)	0.540
Serum chloride, mmol/L	103.01 ± 2.70	102.48 ± 2.15	0.371
hsCRP, mg/L	0.92 (0.48-1.84)	1.20 (0.73-3.45)	0.078
Total cholesterol, mg/dL	152.00 (129.00-176.00)	156.00 (128.25-200.00)	0.573
LDL, mg/dL	83.70 (66.80-102.80)	85.90 (69.63-111.58)	0.624
HDL, mg/dL	49.00 (42.00-63.00)	43.50 (36.75-52.25)	0.072
Triglyceride, mg/dL	105.00 (72.00-154.00)	131.50 (80.75-214.25)	0.222

Fasting Glucose, mg/dL	105.00 (96.80-117.75)	113.00 (99.70-164.00)	0.025
uACR, mg/g	4.69 (0.00-8.22)	92.37 (53.02-127.17)	<0.001
HbA1c, %	5.80 (5.60-6.20)	6.05 (5.68-8.05)	0.039
NT proBNP, pg/mL	147.60 (83.72-366.43)	321.35 (170.18-903.85)	0.005
LVEF, %	52.15 (47.37-57.32)	50.96 (40.23-52.77)	0.022

Data are showed as median (Q1-Q3) or n (%) or mean ± SD. Q1, quartile 1; Q3, quartile 3; SD, standard deviation; kg, kilogram; cm, centimeter; HR, heart rate; bpm, beats per minute; BPs, systolic blood pressure; BPd, diastolic blood pressure; mmHg, millimeters of mercury; eGFR, estimated glomerular filtration rate Chronic Kidney Disease Epidemiology Collaboration *Equation*; mL, milliliter; min, minute; m^2, square meter; WBC, White Blood Cells; thou, thousand; µL, microliter; RBC, Red Blood Cells; mln, million; HGB, hemoglobin; g, gram; dL, deciliter; RDW CV, Red Cell Distribution Width in%; PLT, Platelet Blood Test; MCV, Mean Corpuscular Volume; fL, femtoliter; µg, microgram; mmol, millimole; L, Liter; hsCRP, high-sensitivity C-reactive protein; mg, milligram; LDL, Low-Density Lipoprotein; HDL, High-Density Lipoprotein; uACR, Urine Albumin/Creatinine Ratio; HbA1c, Glycated hemoglobin; NT-proBNP, N-terminal pro-brain natriuretic peptide; pg, picogram; LVEF, Left Ventricle Ejection Fraction.
