# Supplementary material for: Predictors of Long-Term Prognosis Focused on Kidney Function in Patients with Chronic Coronary Syndrome
Source: Diseases. 2026 Feb 19;14(2):78. doi: 10.3390/diseases14020078 (PMC12939443; doi:10.3390/diseases14020078)
Supplement: Supplementary file 1 [file diseases-14-00078-s001.zip › Table S3.pdf]

Table S3 Comparison of groups of patients with absent and present albuminuria only with normal renal function.

Study population (n=212)			
Variables	Subjects with eGFR above or equal to 60 with no albuminuria (n=187)	Subjects with eGFR above or equal to 60 with present albuminuria (n=25)	p Values
Age, years	62.59 ± 7.76	63.40 ±7.67	0.667
Gender, male	145 (77.54)	20 (80)	0.781
Weight, kg	85.57 ± 15.79	87.83 ± 21.12	0.522
Waist circumference, cm	98.52 ± 11.32	102.96 ± 14.27	0.076
HR, bpm	65 (58-71)	73 (65.5-84.5)	<0.001
BPs, mmHg	130 (120.5-144)	137 (121.5-155.5)	0.124
BPd, mmHg	84.10 ± 11.15	87.64 ± 8.79	0.129
eGFR, ml/min/1.73m2	82.16 (72.3-91.97)	85.84 (70.18-95.12)	0.410
WBC, tys/µL	6.3 (5.2-7.5)	6.4 (5.25-7.65)	0.788
RBC, mln/µL	4.76 (4.49-5.04)	4.53 (4.14-4.85)	0.050
HGB, g/dL	14.07 ± 1.1	13.69 ± 1.61	0.128

HCT, %	41.15 (38.8-43.5)	41.2 (38.8-42.65)	0.602
RDW CV, %	14.4 (13.7-15.3)	15.3 (14-16.7)	0.022
PLT, tys/μL	215.5 (187.5-255)	226 (172.5-269.5)	0.759
MCV, fL	86.4 (83.98-89.53)	88.1 (82.45-94.2)	0.233
Serum iron concentration, μg/dL	101.1 (84.3-126.9)	86.3 (56.65-110.1)	0.009
Serum sodium, mmol/L	138.35 (136.78-140.2)	137 (135.1-138.1)	0.003
Serum potassium, mmol/L	4.28 (4.05-4.51)	4.27 (4.11-4.52)	0.650
Serum chloride, mmol/L	103.02 ± 2.83	102.31 ± 3.51	0.253
hsCRP, mg/L	0.99 (0.51-2.18)	1.67 (0.85-3.67)	0.018
Serum calcium, mmol/L	2.41 (2.37-2.47)	2.39 (2.32-2.49)	0.485
Serum inorganic phosphate, mmol/L	3.12 (2.87-3.49)	2.93 (2.75-3.41)	0.324
Total cholesterol, mg/dL	153 (128-178)	157 (126.5-198)	0.355
LDL, mg/dL	83.7 (66.8-104.2)	82.8 (72.65-116.95)	0.634

HDL, mg/dL	49<br>(42-62)	43<br>(33.5-57)	0.064
Triglyceride, mg/dL	99<br>(72-142)	121<br>(86-201.5)	0.109
Fasting glucose, mg/dL	104<br>(96.1-114)	114.5<br>(99.65-176.75)	0.009
uACR, mg/g	4.51<br>(0-7.99)	92.68<br>(63.75-127.53)	<0.001
HbA1c, %	5.8<br>(5.6-6.2)	6.25<br>(5.63-7.28)	0.029
NT proBNP, pg/mL	131.3<br>(76.85-276.43)	240.1<br>(107.98-436.83)	0.022
Pulse pressure, mmHg	46.5<br>(37.25-57)	52<br>(45-59.5)	0.119
Left Ventricle Ejection Fraction, %	52.8<br>(47.8-58.1)	50.7<br>(40.8-52.8)	0.013
Diabetes, n	72 (38.5)	9 (36.0)	0.809
Hypertension, n	152 (81.3)	17 (68.0)	0.623
ACE-inhibitors, n	126 (67.4)	17 (68.0)	0.562
ARB, n	4 (2.1)	0	0.446
MRA, n	51 (27.3)	10 (40.0)	0.233
SGLT2 inhibitors, n	2 (1.1)	0	0.592

Data are shown as median (Q1-Q3) or n (%) or mean ± SD. Q1, quartile 1; Q3, quartile 3; SD, standard deviation; kg, kilogram; cm, centimeter; HR, heart rate; bpm, beats per minute; BPs, systolic blood pressure; BPd, diastolic blood pressure; mmHg,

millimeters of mercury; eGFR, estimated glomerular filtration rate Chronic Kidney Disease Epidemiology Collaboration *Equation*; mL, milliliter; min, minute; m^2, square meter; WBC, White Blood Cells; thou, thousand; µL, microliter; RBC, Red Blood Cells; mln, million; HGB, hemoglobin; g, gram; dL, deciliter; RDW CV, Red Cell Distribution Width in%; PLT, Platelet Blood Test; MCV, Mean Corpuscular Volume; fL, femtoliter; µg, microgram; mmol, millimole; L, Liter; hsCRP, high-sensitivity C-reactive protein; mg, milligram; LDL, Low-Density Lipoprotein; HDL, High-Density Lipoprotein; uACR, Urine Albumin/Creatinine Ratio; HbA1c, Glycated hemoglobin; NT-proBNP, N-terminal pro-brain natriuretic peptide; pg, picogram, LVEF, Left Ventricle Ejection Fraction; ACE-inhibitors, Angiotensin-Converting Enzyme Inhibitors; ARB, Angiotensin II Receptor Blockers; MRA, Mineralocoticoid Receptor Antagonists; SGLT2 inhibitors, Sodium-Glucose Cotransporter-2.
