# Supplementary figures and images for: Predictors of Long-Term Prognosis Focused on Kidney Function in Patients with Chronic Coronary Syndrome
Source: Diseases. 2026 Feb 19;14(2):78. doi: 10.3390/diseases14020078 (PMC12939443; doi:10.3390/diseases14020078)

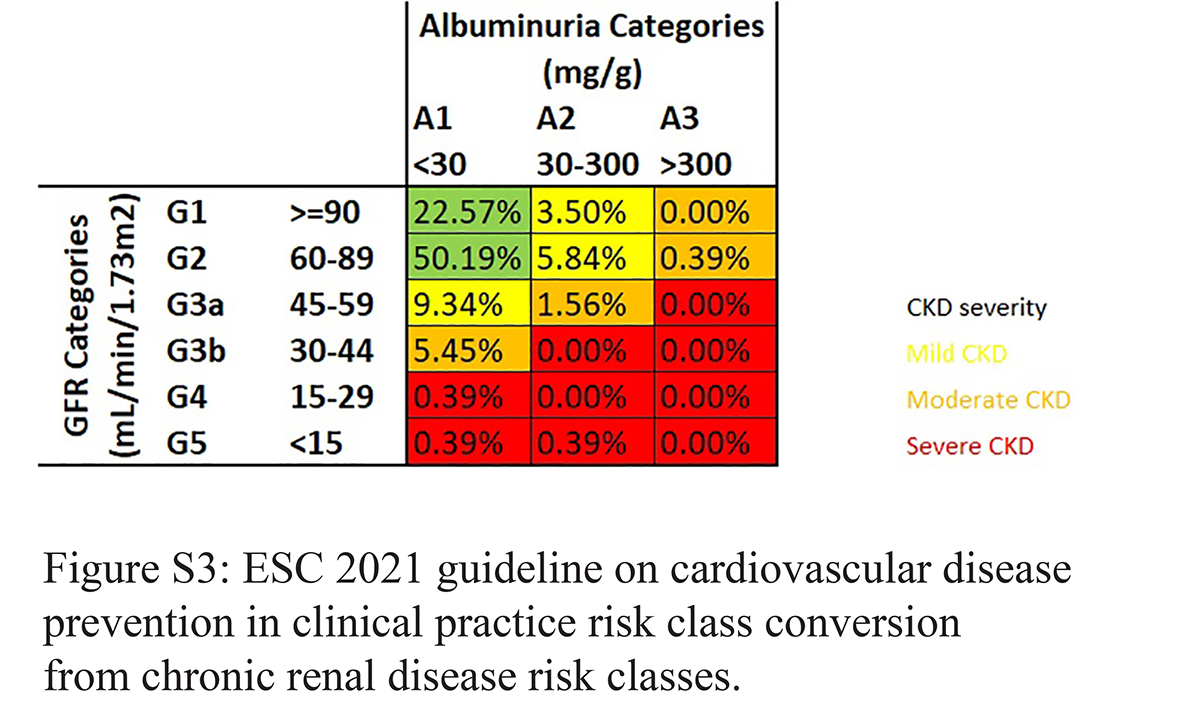

Supplement: Supplementary file 1 [file diseases-14-00078-s001.zip › Figure S3.jpg]

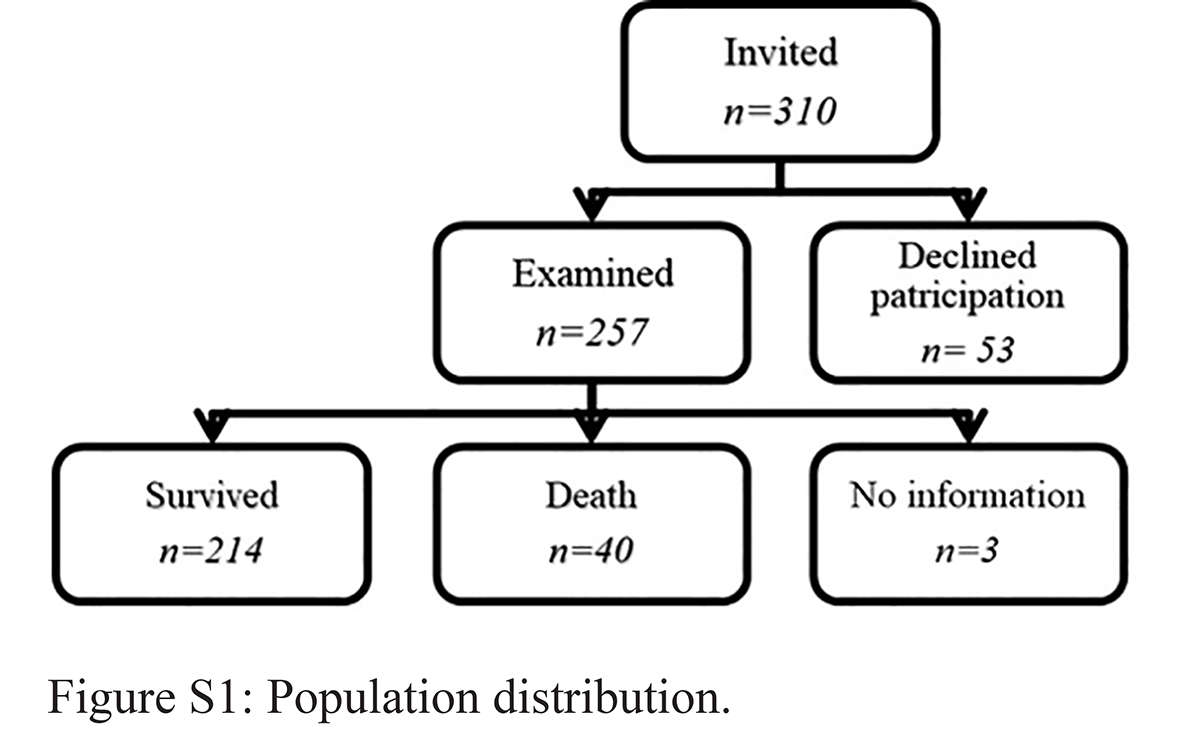

Supplement: Supplementary file 1 [file diseases-14-00078-s001.zip › Figure S1.jpg]

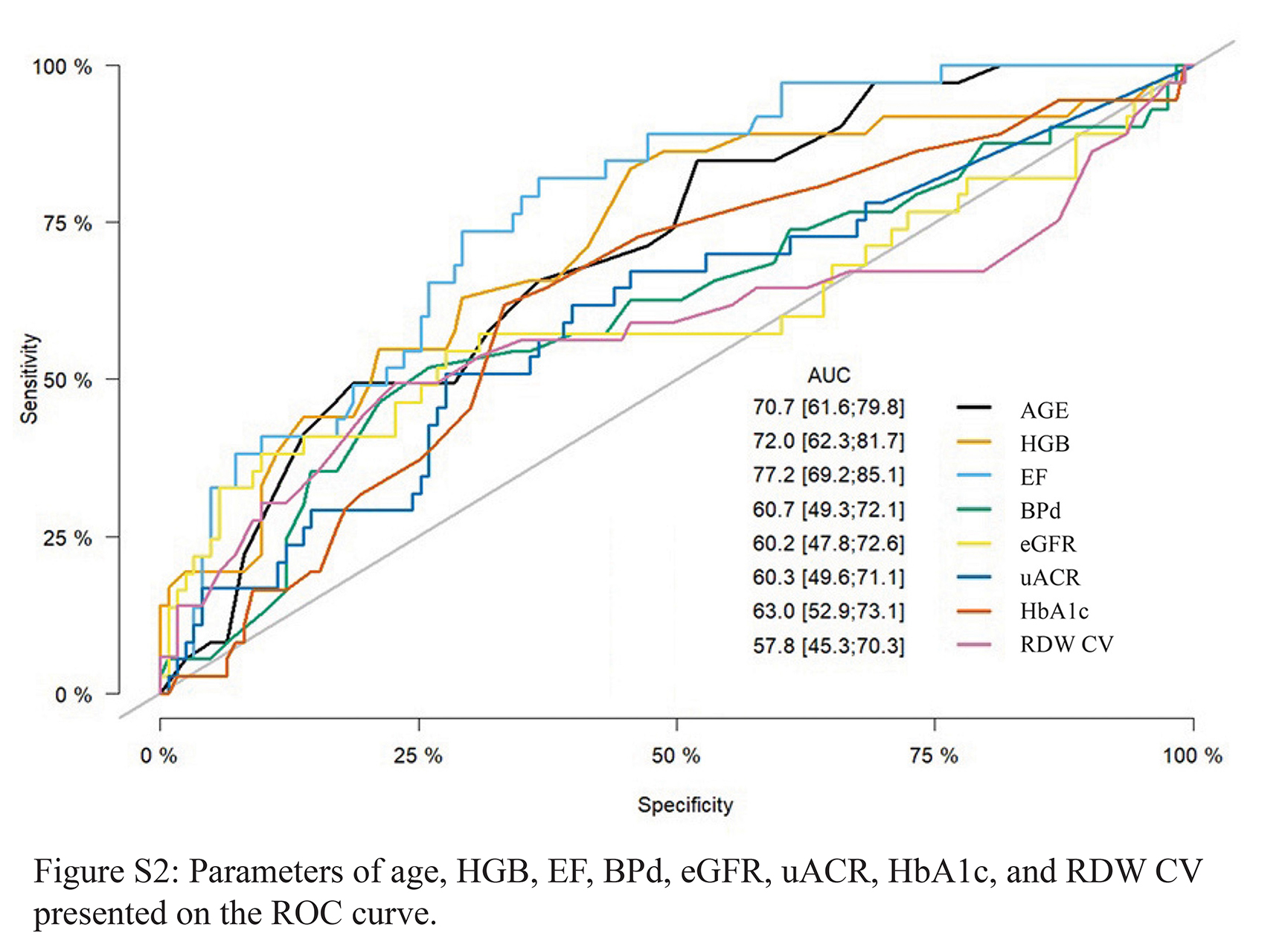

Supplement: Supplementary file 1 [file diseases-14-00078-s001.zip › Figure S2.jpg]
